# Flat-Knitted Double-Tube Structure Capacitive Pressure Sensors Integrated into Fingertips of Fully Fashioned Glove Intended for Therapeutic Use

**DOI:** 10.3390/s24237500

**Published:** 2024-11-25

**Authors:** Susanne Fischer, Carola Böhmer, Shamima Nasrin, Carmen Sachse, Chokri Cherif

**Affiliations:** 1 Institute for Textile Machinery and High Performance Material Technology (ITM), Faculty for Mechanical Science and Engineering, Technische Universität Dresden, 01062 Dresden, Germany; carola.boehmer@tu-dresden.de (C.B.); shamimatex092@gmail.com (S.N.); carmen.sachse@tu-dresden.de (C.S.); chokri.cherif@tu-dresden.de (C.C.); 2 CeTI—Cluster of Excellence, Centre for Tactile Internet with Human-in-the-Loop, Technische Universität Dresden, 01062 Dresden, Germany

**Keywords:** capacitive pressure sensors, flat knitting technology, therapeutic glove, smart textiles, physiotherapy, fully integrated, single process step, sensor-tube structure, double-tube structure

## Abstract

A therapeutic glove, which enables medical non-professionals to perform physiotherapeutic gripping and holding movements on patients, would significantly improve the healthcare situation in physiotherapy. The glove aims to detect the orthogonal pressure load and provide feedback to the user. The use of textile materials for the glove assures comfort and a good fit for the user. This, in turn, implies a textile realization of the sensor system in order to manufacture both the glove and the sensor system in as few process steps as possible, using only one textile manufacturing technique. The flat knitting technology is an obvious choice here. The aim of the study is to develop a textile capacitive pressure sensor that can be integrated into the fingertips of a glove using flat knitting technology and to evaluate its sensor properties with regard to transmission behavior, hysteresis and drift. It was shown that the proposed method of a flat knitting sensor fabrication is suitable for producing both the sensors and the glove in one single process step. In addition, the implementation of an entire glove with integrated pressure sensors, including the necessary electrical connection of the sensor electrodes via knitted conductive paths in three fingers, was successfully demonstrated.

## 1. Introduction

Physiotherapy is one of the most frequently used healthcare services in Germany [1]. Physiotherapy treatments are an important component in providing patients with relief from chronic back pain [2,3,4]; furthermore, they help people who have suffered a stroke to reduce the symptoms of paralysis [5,6,7] and they are an important rehabilitation measure after surgical procedures [8,9,10]. Both the regularity [5,9] and appropriate intensity [5,6] of physiotherapy treatment are crucial for health improvement. However, analyses of the labour market in recent years show a severe shortage of physiotherapists throughout Germany, which remains at a consistently high level [11,12,13,14]. In addition, factors such as an unreasonable travelling distance between the patient and the therapy center, increased care requirements due to the risk of falling or comorbidities of the patient may speak against ambulatory physiotherapy [10]. A shortage of physiotherapists, long distances to practices and patients’ physical limitations can, therefore, be obstacles to optimal treatment with physical therapy. A therapeutic glove that enables medical laypersons (e.g., a family member or acquaintance) to carry out physiotherapeutic gripping and holding movements correctly to perform massage or manual therapy on the patient in their domestic environment would significantly improve the care situation. Furthermore, the glove can be used by the patient themself as a monitoring tool during physiotherapeutic exercises, such as those designed to alleviate paralysis after a stroke, and to restore grasping capabilities of the hand or individual finger. As the glove regularly measures the gripping forces during the exercises, it is uncomplicated to document them on a regular basis. The documentation of the therapy progress in terms of restored grasping would therefore provide an important diagnostic tool to the attending physician.

The aim of the proposed glove is to detect the orthogonal pressure load on the palm and on individual fingers. In order to optimize the comfort and fit of the glove for the user, the glove itself should be made from textile materials. This, in turn, suggests that the sensors also need to be textile-based in order to allow for a simultaneous manufacturing process of the glove and the integrated sensors, with as few process steps as possible, and using only one textile manufacturing process. The flat knitting technology is the obvious choice here. The advantages for the glove itself are the close fit to the body and the increased wearing comfort made possible by fully fashioned knitting. The entire glove is knitted in one single process step and therefore subsequent manufacturing steps, such as joining individually cut pieces with seams, are eliminated. This removes bothersome seams within the glove and has a positive effect on fit and comfort. Subsequent advantages for textile sensor production are, on the one hand, precise positioning within the glove, which is ensured by the knitting program. This allows for an exact positioning of the sensors, even when scaling production, which can be problematic for manual application processes. On the other hand, knitting technology offers the possibility of integrating the sensors directly into the textile. This ensures a robust and accurate fixation in the garment, with no risk of delamination between the textile carrier and the applied textile sensors. These advantages to the production process arise from the possibility of the combined and simultaneous production of the glove and sensors. This, in turn, results in a reduction or even elimination of subsequent manual manufacturing steps and, thus, a reduction of process steps in general.

The functional measuring principles used for textile pressure sensors are primarily resistive or capacitive. Although resistive pressure sensors exhibit a high sensitivity and a wide operating range [15], their disadvantages are a high hysteresis and a large signal drift compared to capacitive sensors [15,16]. Flat-knitted resistive pressure sensors were realized and investigated in [17,18]. In contrast to resistive textile pressure sensors, capacitive textile pressure sensors show a smaller signal drift. However, they possess a lower sensitivity and a limited sensing range, and are susceptible to external interference on the electric field [15,16]. For the described application of the therapeutic glove, which is in use over longer periods of time, the lowest possible drift is crucial in order to be able to reliably assign measured values to the pressure forces applied. For this reason, the capacitive principle is to be preferred for the proposed knitted pressure sensors.

Textile capacitive pressure sensors can be divided into the following three categories in terms of their configuration: in-plane, yarn-based and sandwich configuration [16]. In the in-plane configuration, the two electrodes of the capacitor are arranged in one plane and have an interlocking comb structure. Due to their arrangement in one plane, they are only suitable for pressure applications orthogonal to the surface to a limited extent and are mainly used for expansion applications [19,20]. The yarn-based construction works with a core-sheath structure of the yarn to form a capacitive sensor [15]. The literature describes the yarn-based capacitive pressure sensors as easier to integrate into clothing compared to the sandwich structure, because, due to their yarn shape, they are smaller, easier to process with textile processes and better at adapting to three-dimensional surfaces [15,16]. However, to benefit from these advantages, they have to be manufactured with a diameter small enough to guarantee good processability for further textile integration. With a larger diameter, the possible bending radii are limited, e.g., when the sensor yarn is inserted into the textile in a meandering shape (in order to create a sensor surface that is continuously covered with sensor yarn), which in turn restricts the free placement and the amount of yarn that can be inserted. In addition, the thread-guiding elements of textile machines reach their technical limits above a certain diameter. For this reason, a sandwich construction was chosen, particularly in consideration of the limited space in the fingertip area.

The sandwich structure forms a parallel-plate capacitor and consists of two electrode surfaces which enclose a dielectric layer between them. The sandwich structure can be further subdivided into an all-textile structure and a semi-textile structure. In the all-textile structure, both the electrode surfaces and the dielectric layer are made of textile material, whereas in the semi-textile structure, only one of the two components is made of textile material [16]. Both sandwich structures work according to the same principle of a pressure sensor with the configuration of a plate capacitor. The capacitance can be calculated using the following equation (Equation (1)):

(1) $$C={Ɛ}_{0}{Ɛ}_{r}\frac{A}{d}$$

where Ɛ_0_ is the permittivity of the vacuum, Ɛ*_r_* is the relative permittivity of the dielectric, A is the area of the overlapping electrodes and d is the distance between the electrodes, which is the thickness of the dielectric. The change in capacitance is caused by the change in the distance d between the electrode surfaces. The capacitance increases as the distance decreases and decreases as the distance increases.

Knitted capacitive pressure sensors as all-textile structures with a sandwich structure were successfully implemented in [21,22]. A monofilament thread forms the spacer structure between the knitted electrode surfaces. This spacer structure forms the dielectric in the later sensor, in combination with the enclosed air. The advantage of this implementation is that sensor production is reduced to a single process step, as both the electrode surfaces and the spacer structure can be produced simultaneously on the flat knitting machine. However, the relatively thick layer structure between 25 mm [21] and 6 mm [22,23] is a disadvantage. Even a thickness of 6 mm is unfavorable when positioned at the fingertip, as it significantly impairs the gripping experience. In addition, the compressibility of knitted spacer fabrics is strongly dependent on the material properties of the monofilament itself [21,22] and on their integration into the structure by knitting [24,25]. This, in turn, requires complex investigations to identify the appropriate setting to achieve the desired compressibility for the application, and thus the change in capacitance under compressive load.

Knitted capacitive pressure sensors as a semi-textile structure with a sandwich construction were investigated in [26,27] and successfully attached to the fingertips of a glove. In [26], carbonized knitted layers were used as electrode surfaces and a layer of silicone elastomer was used as a dielectric. The layers were joined together using a sewing process. Furthermore, the electrical contacting of the electrode surfaces and the application of the sensors to the desired position on the glove required additional process steps. In [27], electrically conductive knitted layers, which later form the electrode surfaces of the plate capacitor, were bonded to the dielectric layer in the form of a silicone elastomer using a lamination process. The lamination process used to permanently bond the individual layers to each other is suitable for different combinations of electrode surfaces and dielectric layers. This offers the advantage of an easy adaptation of the sensor properties to the requirements of the application. However, the manufacturing process of the sensor requires several process steps, such as cutting the sensor to size, placing and fixing the sensors onto the fingertip of the glove and the subsequent electrical contacting of the electrode surfaces, as well as the application of conductive tracks.

**Figure 1:**
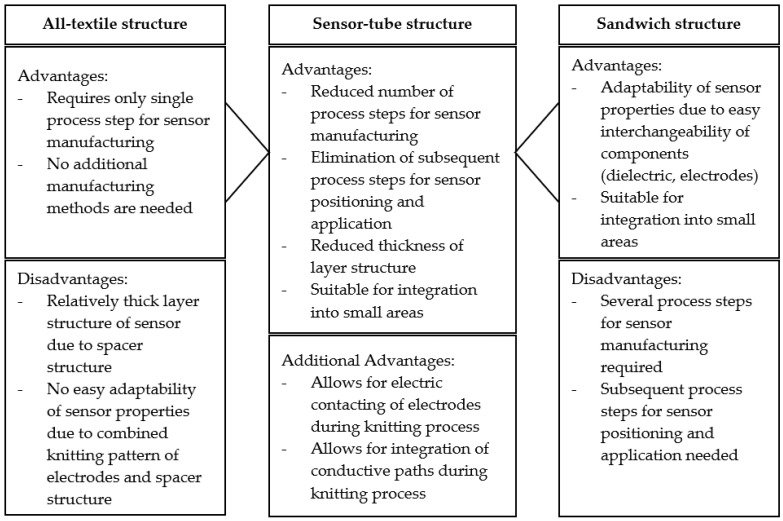
Advantages of the proposed sensor-tube structure.

In the present work, a new manufacturing approach of flat-knitted capacitive pressure sensors, which are suitable for integration even in small areas, such as the fingertips of a glove, is investigated. The intention is to combine the advantages of the knitted all-textile with those of the semi-textile sandwich structure. All components of the glove and the sensors, with the exception of the dielectric layer, are to be manufactured using flat knitting technology. The dielectric layer is to be inserted between the knitted electrode surfaces during the knitting process. This should enable the sensors to be integrated directly into the glove during the manufacturing process and eliminate all subsequent process steps. The thickness of the layer structure can be reduced due to the free choice of dielectric thickness. In addition, a sensor manufactured in this way offers the possibility of implementing the electrical contacting and the conductive paths directly during the knitting process of the glove as well. The advantages of the proposed sensor-tube structure are summarized in Figure 1.

The objectives of this study are as follows:

- Development of a flat knitting manufacturing process for capacitive pressure sensors with a sandwich structure, which requires neither subsequent process steps for sensor production nor for sensor application.
- Evaluation of the sensor with regard to transmission behavior, drift and hysteresis under pressure load.
- Integration of the developed sensors into a complete glove as a fully fashioned article and realization of this glove as a proof of concept of the developed manufacturing process.

## 2. Materials and Methods

In the first stage, the knitting process for manufacturing the pressure sensors was developed and samples of individual fingers with the integrated pressure sensors were knitted. These samples were evaluated with regard to transmission behavior, drift and hysteresis under pressure load. In the next stage the knitting program was extended so that the sensors were electrically contacted to knitted conductive paths directly during the knitting process. Finally, the pressure sensors were integrated at the positions of the thumb, index finger and middle finger of a knitted glove, and the glove was implemented and knitted as a fully fashioned article. In the following, the materials used are listed, the knitting process for manufacturing the fingers with integrated pressure sensors is described, the test setup and procedure of the pressure test are described and the expansion of the fingers into a fully fashioned glove is explained.

### 2.1. Yarn and Dielectric Materials

The conductive and non-conductive materials used for the finger samples are listed in Table 1. A non-conductive yarn, the double-covered elastic yarn 100C from the supplier Julius Zorn GmbH (Aichach, Germany), with a final yarn count of 170 dtex, was used as the base yarn for the finger.

The electrically conductive yarn Silver-tech+ 150 from the supplier Amann & Söhne GmbH & Co. KG (Bönnigheim, Germany) was used for knitting the electrode surfaces and the conductive paths. The yarn has a resistance of less than 300 Ω/m and a yarn count of 220 dtex. The Silver-tech+ 150 was plated with the covered elastic yarn in the area of the electrodes.

The dielectric silicone insert was prepared by casting silicone rubber (silicone rubber type 2) from the supplier TFC Troll Factory (Riede, Germany) in a prepared 3D-printed mold in order to obtain the appropriate thicknesses of 1 mm and 2 mm. According to the manufacturer, the cured silicone has a Shore hardness of 35 Shore A. The final thickness of the dielectric was determined after curing using an analog caliper gauge with a measuring accuracy of 0.05 mm. A thickness of 1.0 mm was measured for the thinner silicone insert and a thickness of 2.0 mm was measured for the thicker silicone insert.

### 2.2. Knitting Process

**Figure 2:**
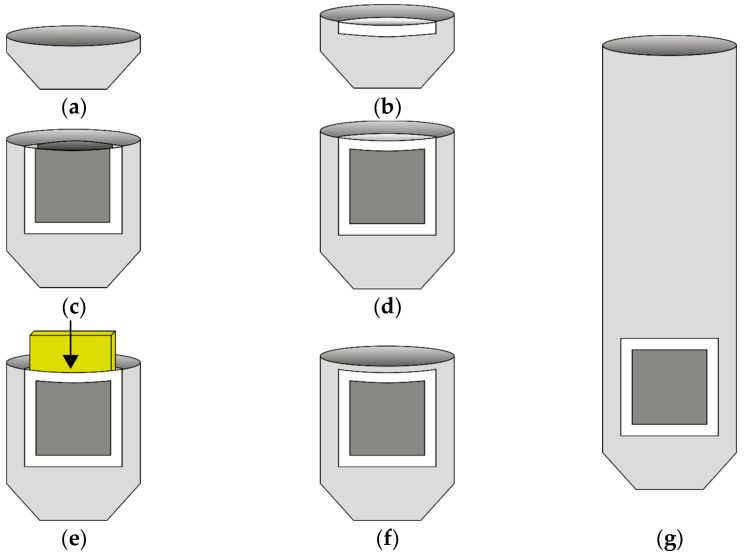
Knitting process of the samples. (**a**) Base structure starting at the fingertip; (**b**) double-tube structure starting with insulating protective rows (white); (**c**) electrode surfaces (dark grey) with insulating protective rows at the sides (white); (**d**) insulating protective rows at the end of the electrode surfaces (white), wherein the double-tube structure is now finished; (**e**) inserting the dielectric layer (displayed in yellow); (**f**) the pocket is closed by knitting; (**g**) the remaining finger is completed as a simple tubular structure.

**Figure 3:**
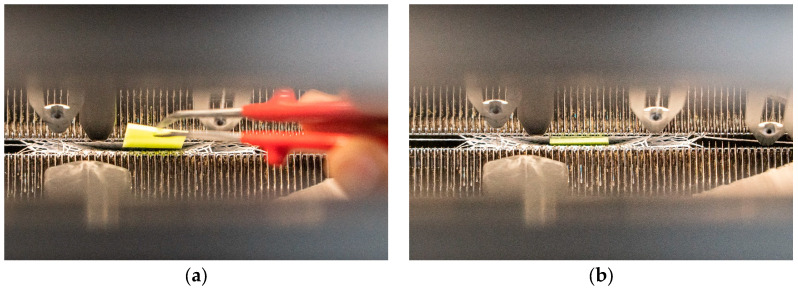

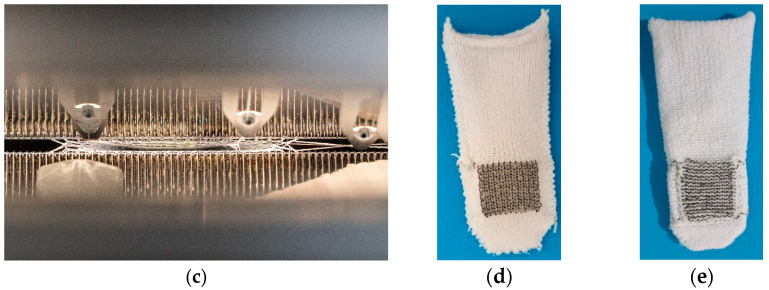
Integrating the dielectric. (**a**) Inserting the dielectric layer; (**b**) dielectric layer is positioned in the knitted sensor pocket; (**c**) closing the sensor pocket; (**d**) right side/outside and (**e**) left side/inside of knitted finger sample with integrated sensor.

The samples for the pressure test were designed as individual fingers with integrated sensors at the fingertips. The knitting program for the samples was created using the Stoll M1Plus software V7.6 from KARL MAYER Holding SE & Co. KG (Obertshausen Germany). The samples were knitted on the Stoll ADF 530-32 BW knit and wear (E14 machine gauge) flat knitting machine from KARL MAYER Holding SE & Co. KG. The fingers were knitted starting at the fingertip and ending at the base of the finger. The areas of the samples can be divided into an electrically non-conductive base structure, two electrically conductive electrode surfaces and electrically insulating protective rows surrounding the electrode surfaces. In the area of the sensor, which comprises the electrode surfaces and the protective rows, the samples are three-layered; in the area of the base structure they are two-layered. The samples therefore have a double-tube structure in the area of the sensor, wherein one tube forms the finger in order to be worn and one tube is for inserting the dielectric layer. This second tube is formed as a pocket, which is closed by knitting after the dielectric layer has been inserted. The electrode surfaces each comprise 10 knitted wales and have a size of 1.5 cm × 1.5 cm, with 11 courses and 7 wales per cm. At the top and bottom of the pocket there are two courses of electrically insulating yarn, which serve as protective rows to prevent electric contact between the two electrode surfaces. Furthermore, for reasons of insulation, there are two wales of electrically insulating yarn on each side of the pocket. The finger was realized as a 1 × 1 tubular structure in both the front and rear needle bed. This makes it possible to form an intermediate separate third layer by regularly transferring loops between the unassigned needles of the front and rear needle bed. The knitting process is shown schematically in Figure 2. First, the basic structure is formed as a simple tube with non-conductive yarn, starting at the fingertip (cf. Figure 2a). From the beginning of the sensor area, the finger is manufactured as a double-tube structure, starting with the insulating protective courses (cf. Figure 2b). The electrode surfaces, which are bordered on the sides by insulating wales, begin after the protective courses (cf. Figure 2c). At the end of the electrode surfaces there are again two insulating protective courses (cf. Figure 2d). The pocket with the parallel electrode surfaces is now completed. In the next step, the dielectric layer is inserted into the pocket (cf. Figure 2e) and the pocket is closed by knitting (cf. Figure 2f). The remaining finger can now be finished with a simple tubular structure (cf. Figure 2g). The process of inserting the dielectric layer is shown in Figure 3. After the pocket with the integrated electrode surfaces and protective rows has been fully formed, the knitting process is paused. The dielectric layer is inserted manually (cf. Figure 3a) and positioned in the pocket (cf. Figure 3b). The pocket is then closed by transferring the loops to the rear needle bed (cf. Figure 3c) and by knitting subsequent courses. In Figure 3d,e, a finished sample of a finger with an integrated sensor is shown. Five samples were manufactured for each dielectric variant, as follows: five fingers with a dielectric thickness of 1 mm and five samples with a dielectric thickness of 2 mm.

### 2.3. Cyclic Pressure Test

**Figure 4:**
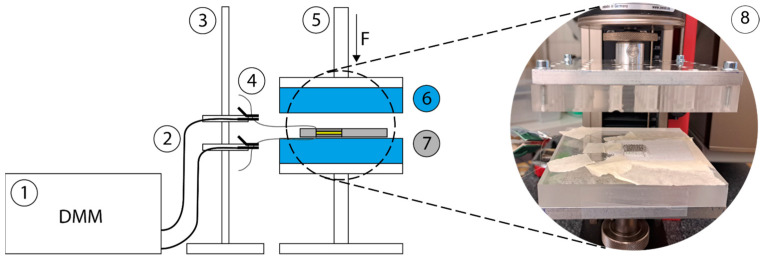
Test setup for cyclic pressure loading with synchronized capacitance measurement; (**1**) digital multimeter, (**2**) cables, (**3**) stand as cable holder, (**4**) clamps, (**5**) universal testing machine for cyclic pressure loading, (**6**) acrylic plates, (**7**) sample to be tested, (**8**) test sample cut open and fixed under testing machine.

Cyclic pressure loading and unloading by the universal testing machine from ZwickRoell GmbH & Co. KG (Ulm, Germany), with a synchronous measurement of the capacitance by the digital multimeter model Keithley DMM6500 from Tektronix, Inc. (Beaverton, OR, USA), were used to characterize the samples. The test setup is shown in Figure 4. The pressure plates with a size of 110 × 110 mm were covered with 14.5 mm thick acrylic plates to insulate the metal plates of the testing machine, and also to minimize parasitic capacitances. The test samples were knitted as a closed tube in order to demonstrate the feasibility of fully fashioned knitting. During gripping movements of a worn finger, the pressure is only applied onto one knitted layer, which is the one with the integrated sensor. If the finger were to be placed under the pressure plates as a knitted tube, the test pressure would act jointly on both knitted layers, as well as the back and front finger. In order to create conditions as close to reality as possible for the pressure test, the test sample was cut open lengthwise at the center back and fixed to the pressure plate along its edges with adhesive tape (cf. Figure 4 (8)).

In order to obtain a good estimate of the measurement quality of the sensors, the compressive forces were set in the range of 0.5–10 N. The sensor surfaces have a size of 1.5 cm × 1.5 cm, resulting in a pressure force of 2.2 kPa–44.4 kPa. This largely corresponds to the range proposed in [26] for pressure to meet gentle touch (low-pressure regimes: 0–10 kPa) and object manipulation (medium-pressure regimes: 10–100 kPa). The test parameters are listed in Table 2.

**Figure 5:**
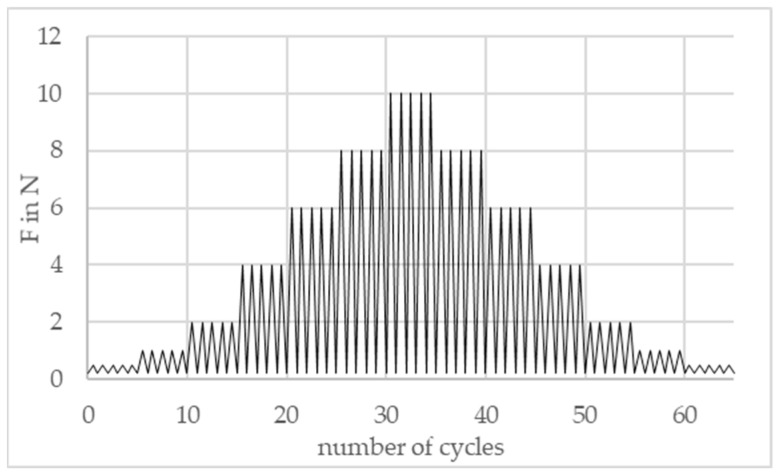
Testing procedure.

At a test speed of 3 mm/min, the test samples are loaded and unloaded with compressive forces of 0.5 N–10 N in stages of 5 cycles each. This is conducted with both increasing and decreasing compressive forces. Figure 5 shows the testing procedure.

### 2.4. Methods of Analysis

The analysis was carried out using the Matlab program R2022b from The MathWorks, Inc. (Natick, MA, USA) The recorded capacitance values showed a high level of noise. For this reason, the Butterworth filter provided in Matlab was used to reduce the noise. The data processed by the filter were used as a basis to determine the transmission behavior, hysteresis and drift.

**Figure 6:**
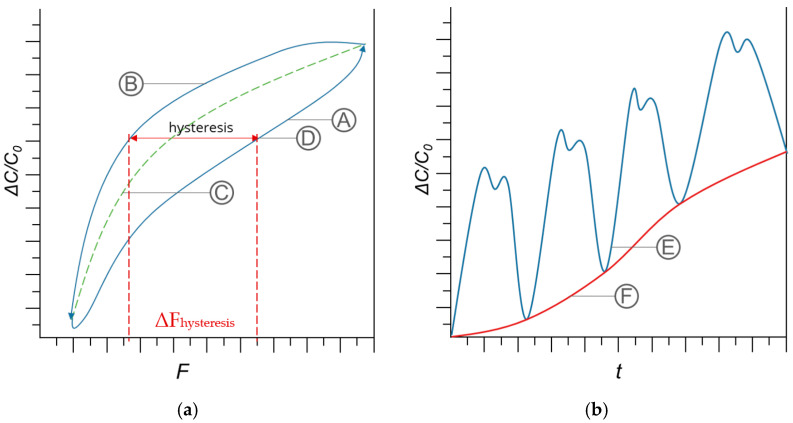
Transmission behavior, hysteresis and drift. (**a**) Transmission behavior and hysteresis; (A) loading; (B) unloading; (C) mean transmission curve; (D) hysteresis. (**b**) Drift; (E) capacitance data; (F) drift curve.

The transmission behavior is defined by the relative change in capacitance (ΔC/C_0_) over the applied force (F) (cf. Figure 6a). A different transmission behavior is to be expected here for the loading and unloading process of the sensor. The averaged value of the loading and unloading curve results in the mean transmission curve. The hysteresis behaviour of the sensor describes the deviation between the sensor´s transmission behaviour of the loading and unloading curve. The strongest deviation in relation to the force applied between loading and unloading is determined and specified in this work as the hysteresis value ΔF_hysteresis_. This is conducted in accordance with [28]. The drift is determined by means of a drift curve. This curve describes the course of the measured capacitance values over time if no additional force is applied apart from the pre-load of 0.2 N (cf. Figure 6b).

### 2.5. Development of the Full Glove

**Figure 7:**
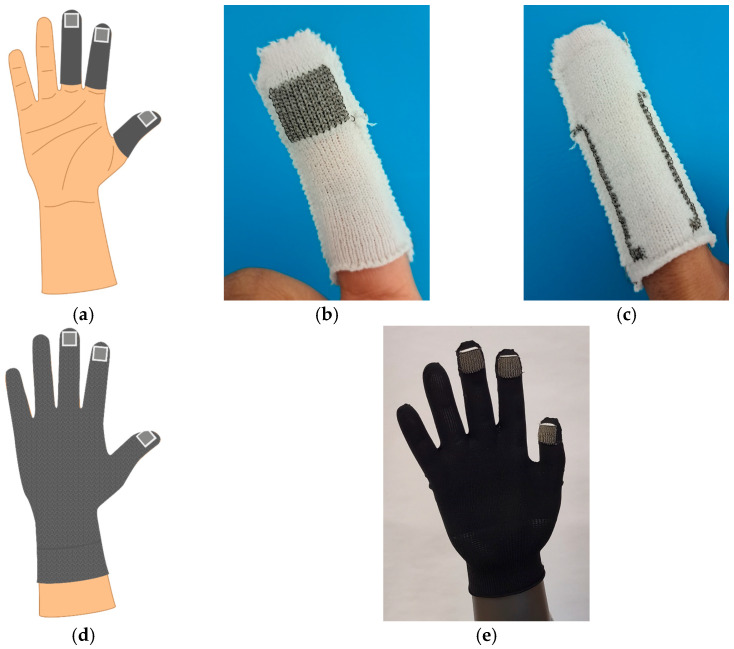
Development of full glove. (**a**) Schematic of the sensor positions; (**b**) front of finger with integrated conductive paths; (**c**) back of finger with integrated conductive paths; (**d**) schematic of full glove; (**e**) knitted glove as fully fashioned article including sensors and conductive paths.

The manufactured test samples for the pressure tests were deliberately manufactured without conductive paths in order to exclude possible influences on the capacitance measurement. The next development stage was including knitted conductive paths into the knitting program of the fingers. To prevent the conductive paths from running through the gripping area, they were placed on the back of the finger. The respective yarn guide for knitting the electrode surface was also used for knitting the corresponding conductive path. This ensures that the electrode surface and the associated conductive path can be knitted with one continuous yarn without interruption, thereby guaranteeing reliable electrical contact between the two. Figure 7b,c shows the results.

For the further development of the full glove (cf. Figure 7d), the base yarn was changed to a covered elastic yarn from Jörg Lederer GmbH (Amstetten, Germany) in black due to availability and optical reasons. The material of the yarn is PA6/Lycra, with a final yarn count of 180 dtex (130/78f18/1). Three fingers of the glove—the thumb, index and middle finger—were equipped with capacitive pressure sensors. The knitted glove as a fully fashioned article is shown in Figure 7e.

## 3. Results

**Figure 8:**
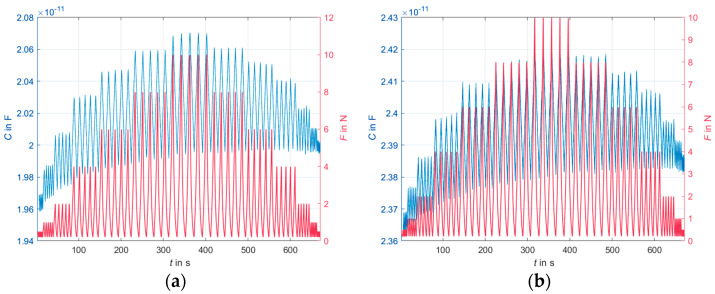
Measured capacitance and the applied forces in absolute values over time; (**a**) dielectric of 1 mm thickness; (**b**) dielectric of 2 mm thickness.

Figure 8 shows the measured capacitance and the applied forces in absolute values over time.

**Figure 9:**
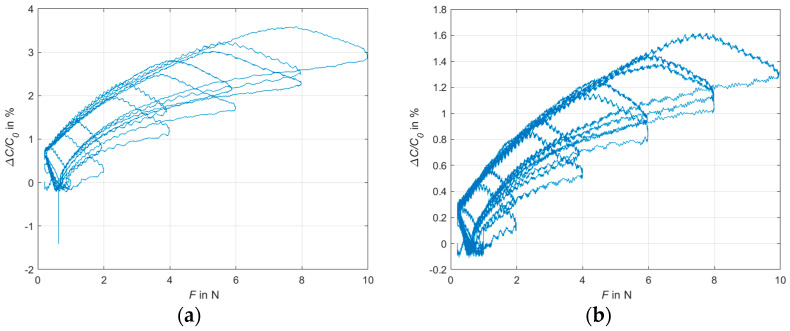
Transmission behavior of the sensors over all cycles. (**a**) Dielectric of 1 mm thickness; (**b**) dielectric of 2 mm thickness.

The transmission behavior, the drift and the hysteresis under the pressure load were determined for the sensors with the dielectric of 1 mm, as well as 2 mm thickness. Figure 9 shows the transmission behavior of the sensors with a 1 mm and 2 mm dielectric over all tested cycles.

**Figure 10:**
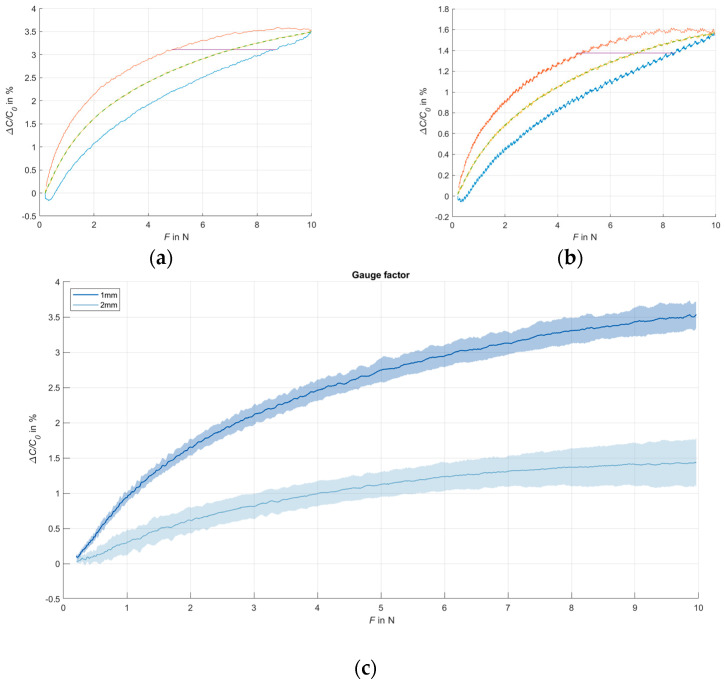
Transmission behavior of the sensors and resulting hysteresis; 5 cycles with 10 N. (**a**) Transmission behavior of dielectric of 1 mm thickness and resulting hysteresis (transmission behavior loading phase displayed in blue, transmission behavior unloading phase displayed in red, mean transmission curve displayed in yellow); (**b**) transmission behavior of dielectric of 2 mm thickness and resulting hysteresis (transmission behavior loading phase displayed in blue, transmission behavior unloading phase displayed in red, mean transmission curve displayed in yellow); (**c**) resulting gauge factor and standard deviation of dielectrics of 1 mm and 2 mm.

The five cycles with 10 N were used for a more detailed sensor analysis. The resulting transmission behavior is shown in Figure 10a,b in relative values, as well as the resulting maximum hysteresis. The resulting gauge factor is shown in Figure 10c.

**Figure 11:**
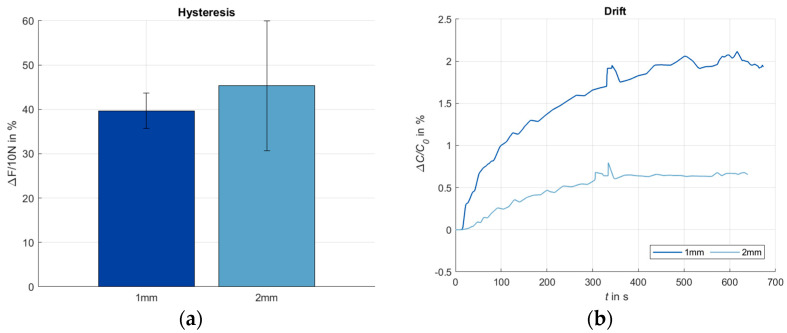
Hysteresis and drift. (**a**) Hysteresis values and standard deviation of the sensors with dielectrics of 1 mm and 2 mm thickness; 5 cycles with 10 N; (**b**) drift of the sensors with a dielectric of 1 mm and 2 mm thickness; all cycles.

The calculated values for hysteresis are shown in Figure 11a. The resulting drift is shown in Figure 11b. The drift was determined over all cycles.

## 4. Discussion

Developed manufacturing process

The developed manufacturing process for the knitted capacitive pressure sensors proved to be very suitable. It was possible to implement the fingers as a double-tube structure. This enabled the additional integration of a pocket, in which the dielectric layer was inserted. It was also possible to integrate the knitted electrodes into the sides of the pocket. The knitting process only needs to be paused to insert the dielectric layer. The process neither requires subsequent process steps for sensor production nor sensor application. The manual feeding of the dielectric layer—regardless of a dielectric thickness of 1 mm or 2 mm—and the subsequent closing of the pocket by machine knitting proved to be unproblematic. This provides a great deal of flexibility for future modifications regarding the choice of the dielectric material. It is also highly probable that the process could be adapted to automatic feeding. The insulation of the electrode surfaces from each other by knitted protective rows has proven to be effective. The pressure test has shown that there is no electrical contact between the electrode surfaces, even under a pressure load of 10 N.

Based on the magnitude of the measured capacitance (the measured values are in the range of 20 pF—cf. Figure 8) and taking into account the size of the overlapping electrode surfaces and the thickness of the dielectric, it can be assumed that part of the measured capacitance is due to influences of the measurement cables and/or the environment, e.g., the universal testing machine used for the pressure tests. The acrylic plates may not provide a sufficient distance between the sensor samples and the metal plates of the universal testing machine. Additional passive shielding is recommended for future measurements. In addition, the capacitance of the measuring cables should be measured separately and taken into account for future measurements. Therefore, the present measurement results should be considered in light of the influence of a systematic error, but nevertheless, they provide a good basis for evaluating the sensor behavior.

Transmission behavior

Figure 9 and Figure 10 show that there is a correlation between the magnitude of the applied force and the measured capacitance over the entire tested range of 0.5–10 N. In the lower pressure range, the sensitivity of the sensor is higher than in the higher pressure range; however, there is still a correlation between capacitance change and applied force, even in the higher pressure range tested, which leads to the conclusion that the sensor’s working range is not limited at 10 N. Comparing the cycles of the different force levels with each other in Figure 9, it is noticeable that the change in capacitance in relation to the applied force differs more in the loading phases than in the unloading phases. In Figure 10, solely the transmission behavior of the cycles with 10 N are shown. The sensors with the dielectric of 1 mm thickness have a relative change in capacitance of 3.5% at 10 N, while the dielectric of 2 mm has a change of only 1.6%. This is due to the smaller distance between the electrode surfaces for the dielectric of 1 mm. Because of the higher relative change in capacitance, the dielectric of 1 mm is preferable to 2 mm for the further development of the glove. In addition, the wearer feels more comfortable with a thickness of 1 mm than with 2 mm. The thinner layer structure ensures a higher remaining sensitivity during gripping movements in the fingertips.

Hysteresis

The most significant hysteresis for the sensors with the dielectric of 1 mm thickness appears between 4.9 N and 8.7 N, with the dielectric of 2 mm thickness between 4.7 N and 8.5 N (cf. Figure 10). This results in values for ΔF_hysteresis_ of 39.7% (standard deviation 4.0%) and 45.3% (standard deviation 14.6%), respectively (cf. Figure 11a). This hysteresis, which results from the different sensor behaviors during loading and unloading, significantly impairs the sensor accuracy. To increase the sensor accuracy, a separate evaluation for loading and unloading phases is recommended.

Drift

The drift of the measured capacitance values over all 65 tested cycles is for the dielectric of 1 mm thickness, which is less than 2.15%, while for the dielectric of 2 mm thickness, it is less than 0.8% (cf. Figure 11b). Despite the low percentage values, the drift has to be considered in relation to the transmission behavior. The sensors with the dielectric of 1 mm thickness have a relative change in capacitance of 3.5% at 10 N, while the dielectric of 2 mm has a change of 1.6%. Based on these results, the drift must be taken into account when determining the pressure load via the transmission behavior of the sensor. In this work, the determination of the drift was only a first indication to be able to estimate the dimensions. A more in-depth investigation of the causes, the long-term behavior and possible strategies to reduce the drift are necessary.

Integration of sensors in fully fashioned glove

The further development of the manufacturing process has shown that the developed knitting process of the sensors is even applicable for the production of a fully fashioned glove with integrated sensors. It has not yet been tested under pressure, as this was primarily a proof of concept for transferability; however, it can be assumed that the sensors behave comparably to the tested samples, as the knitting process of the sensors was retained. The electrical connection of the electrode surfaces via integrated flat-knitted conductive paths was also successfully implemented.

Further steps concerning the manufacturing process of the glove are the development and integration of a preferably knitted insulation of the inner electrode surfaces to the body. At the moment, these electrode surfaces are still in direct contact with the body, and this would affect the capacitance measurement. In addition, electrical insulation of the conductive paths to the body is also functionally relevant.

## 5. Conclusions

In the present work, a new manufacturing approach—the sensor-tube structure—of flat-knitted capacitive pressure sensors, which combines the advantages of the knitted all-textile structure and the semi-textile sandwich structure, was investigated.

The results of the study are as follows:

- This paper presents a new approach to the manufacturing of flat-knitted capacitive pressure sensors. All components of the sensors, with the exception of the dielectric layer, can be manufactured using flat knitting technology. The dielectric layer is to be inserted between the knitted electrode surfaces during the knitting process. The sensors can be integrated directly into the glove during the manufacturing process, forming a double-tube structure. The developed process neither requires subsequent process steps for sensor production nor sensor application. In addition, the process allows for electric contacting of the electrodes and for integration of conductive paths during the knitting process.
- The measurements under the pressure load of the sensors showed promising results. The transmission behavior of the sensors shows a correlation between the applied compressive force and the measured capacitance over the entire tested pressure range. This indicates that the sensor’s working range is not limited to the range of 0.5–10 N, which corresponds to a pressure force of 2.2 kPa–44.4 kPa. The sensors with the dielectric of 1 mm thickness show a higher relative change in capacitance than the dielectric of 2 mm, and are therefore preferable for the further development of the glove. Due to the pronounced hysteresis loop between the loading and unloading curve, a separate evaluation for loading and unloading phases is recommended to increase the sensor accuracy for application. The evaluation of the drift has shown that the drift has to be taken into account when determining the pressure load via the transmission behavior of the sensor. Further investigations are required for this.
- The developed sensors were successfully integrated into a complete glove by flat knitting technology. This served as proof of concept and demonstrated that the developed manufacturing process is suitable to produce fully fashioned articles with integrated knitted capacitive pressure sensors in one process step.

Despite the promising results, further investigations are needed for an in-depth sensor characterization and analysis of the working mechanism, such as the following:

- The cause for hysteresis and drift can either be the knitted structure itself, i.e., the knitted electrode surfaces; the dielectric used, i.e., the silicone insert; or a combination of both components. Further research is necessary to understand the working mechanism and the interaction of the components with each other to be able to allocate the share of the effects to the respective partners.
- The results have shown that the sensor’s working range is not limited to 10 N. In further tests, the applied force in cyclic pressure tests is to be increased to 50 N, which corresponds to an applied pressure of 222 kPa for the sensors.
- The longer-term behavior of the sensors under a repeated pressure load is also to be investigated more closely in terms of repeatability.
- Furthermore, the integration of multiple sensors into the fingers, as well as the palm of a glove, is planned for testing the sensor function during specific gripping and handling movements.

## Figures and Tables

**Table 1:** Used conductive and non-conductive yarn materials and dielectric material for finger samples with integrated knitted pressure sensor.

Material	Composition	Yarn Count/Thickness	Supplier	Used for
Covered elastic yarn/100C	Lycra/PA6	17 tex	Julius Zorn GmbH	Base yarn
Silver-tech+ 150	Silver-covered PA/PES	22 tex	Amann Group	Knitted electrode
Silicone inserts	Silicone rubber (type 2)	1 mm 2 mm	TFC Troll Factory	Dielectric layer

**Table 2:** Test parameter for universal testing machine and digital multimeter.

Test Instrument	Parameter	Value
Tensile tester Universal Testing Machine zwickiLine ZwickRoell	Pre-load	0.2 N
	Testing speed	3 mm/min
	Test loads	0.5 N, 1 N, 2 N, 4 N, 6 N, 8 N, 10 N
	Cycles per test load	5
Digital Multimeter DMM6500 Keithley Tektronix	Measurement method	Constant current slope measurement
	Sampling rate	100 Hz
	Measurement Range	1 nF (automatic mode)
	Resolution	0.1 pF
	Capacitance accuracy ± (% of reading + % of range)	0.80 + 0.50

## Data Availability

The data presented in this study are available on request from the corresponding author.
